# Phytochemicals from *Ruta graveolens* Activate TAS2R Bitter Taste Receptors and TRP Channels Involved in Gustation and Nociception

**DOI:** 10.3390/molecules201018907

**Published:** 2015-10-16

**Authors:** Giuseppe Mancuso, Gigliola Borgonovo, Leonardo Scaglioni, Angela Bassoli

**Affiliations:** DeFENS—Department of Food, Environmental and Nutritional Sciences, University of Milan, via Celoria 2, Milano I-20133, Italy; E-Mails: manq.hsp@libero.it (G.M.); gigliola.borgonovo@unimi.it (G.B.); leonardo.scaglioni@unimi.it (L.S.)

**Keywords:** *Ruta graveolens*, bitter taste, TAS2Rs receptors, chemesthesis, TRP channels

## Abstract

*Ruta graveolens* (rue) is a spontaneous plant in the Mediterranean area with a strong aroma and a very intense bitter taste, used in gastronomy and in folk medicine. From the leaves, stems and fruits of rue, we isolated rutin, rutamarin, three furanocoumarins, two quinolinic alkaloids, a dicoumarin and two long chain ketones. Bitter taste and chemesthetic properties have been evaluated by *in vitro* assays with twenty receptors of the TAS2R family and four TRP ion channels involved in gustation and nociception. Among the alkaloids, skimmianine was active as a specific agonist of T2R14, whereas kokusaginin did not activate any of the tested receptors. The furanocoumarins activates TAS2R10, 14, and 49 with different degrees of selectivity, as well as the TRPA1 somatosensory ion channel. Rutamarin is an agonist of TRPM5 and TRPV1 and a strong antagonist of TRPM8 ion channels.

## 1. Introduction

### 1.1. Taste Chemoreception and Bioactive Compounds

The investigation of bioactive compounds from traditional food and food plants has always been a topic of interest for food chemistry; on the other hand, the taste profile of a plant has for a long time been a topic of interest mainly for sensory analysis and consumer science. In recent years, the idea of connecting the taste properties of a plant or an ingredient at a molecular level to the presence of compounds with potential benefits for health and nutrition has received growing attention [1,2,3,4]. A major advance in this field has been contributed by knowledge about the functions of chemosensory (olfaction, taste and somatosensory) receptors in detecting active compounds in food and the environment. These receptors constitute an extraordinary apparatus, not only in food selection, but also in communication; in fact, they not only exert a positive or negative selection in regulating food intake, but are also used to decode social and environmental signals among plants, invertebrates and animals.

In this context, two particular classes of receptors have received our attention: the bitter taste receptors of the TAS2R family and the ion channels belonging to the transient receptor potential (TRP) family of chemesthetic receptors.

The bitter sensation is mediated by a wide spectrum of GPCRs that are part of the taste 2 receptor family (TAS2Rs), which comprises twenty-five different receptors in humans, each displaying its own specificity [4]. A number of papers describe the importance of mapping dietary bitter compounds in order to understand their role and mechanisms [5,6].

TRP ion channels are a large family of receptors involved in sensing the external environment, including vision, olfaction, thermosensation and many other kinds of sensory modalities [7,8]. Some of them contribute to the perception of so-called somatosensory sensations, such as pungency, hotness or coolness, generated by many spices and food plants in many organisms, including all metazoan organisms and man.

A common function of these systems is that of warning against dangerous biological and chemical agents in the environment, constituting an array of defense mechanisms, also defined as the “chemofensor complex” [9]. Taste can be used as a tool to detect interesting bioactive compounds by means of so-called “taste guided” analysis.

Many food plants used in traditional cooking and in folk medicine have a distinct bitter taste and/or chemesthetic features; among them, we decided to investigate *Ruta graveolens*.

### 1.2. Ruta graveolens, Medicinal Uses and Taste

*Ruta graveolens* (Rutaceae), commonly known as rue, has been well known since ancient times as a culinary and medicinal plant. Its name derives from the ancient Greek ῥυτἠ from the verb ῥυομαι = to save, to protect, probably in reference to its capacity to preserve its leaves for a very long time and to protect health. The adjective *graveolens* refers instead to the smell of the leaves, which have a strong balsamic odor, sometimes perceived as pungent and/or unpleasant [10]. The phytochemical profile of rue is quite complex and includes psoralens, furocoumarins, alkaloids, long chain ketones and other compounds [11,12].

The uses of rue in cuisine and in medicine are well described [13]. In gastronomy, rue is used for its typical pungent aroma and the very bitter taste of its aerial parts, mainly to aromatize some meat and egg preparations and to prepare a traditional alcoholic beverage (grappa alla ruta) popular in Northern Italy and Croatia.

Much more is known about its medicinal uses. The plant has, in fact, been known in folk medicine since ancient times and is currently used for the treatment of such varied disorders as pain, rheumatism, eye problems and dermatitis. Extracts of rue have been used as antidotes for some snake and scorpion venoms [14] and to treat many infections and inflammation [15]. Rue extracts have been shown to have potent anti-cancer activity, exhibited through strong anti-proliferative and anti-survival effects on cancer cells [16].

Psoralenes, among the main constituents of rue, are known for their photosensitization effects, which can produce a very strong undesirable syndrome in the form of photodermatitis. They are also used for therapeutic purposes in photochemotherapy to treat cutaneous T-cell lymphoma [17] and granuloma annulare [18]. Very recently, it has been reported that (+)-rutamarin is a dual inducer of both GLUT4 translocation and expression and therefore ameliorates glucose homeostasis in insulin-resistant mice [19]. The alkaloid skimmianine has been demonstrated to have anti-inflammatory activity [20]. The antinociceptive activity of rue has been studied in mice, showing a mechanism mediated by opioidergic and alpha-adrenergic receptors, but not by serotonergic receptors [21].

*Ruta graveolens* extracts and essential oils also have well-documented applications in agriculture and crop protection. They are used as fungicides [22,23] and have a phytotoxic activity [11]. Moreover, rue essential oils have a repellent activity against many animals and invertebrates. Their activity has also been tested on *Plasmodium falciparum* [24] and insects [25,26]. The activity of 2-undecanone, the main constituent of rue essential oil, as a repellent has been extensively described against other animals as varied as bugs, voles, amphibian and dogs, although there have been no extensive studies on its molecular mechanisms of action.

### 1.3. Aim of This Work

**Figure 1:**
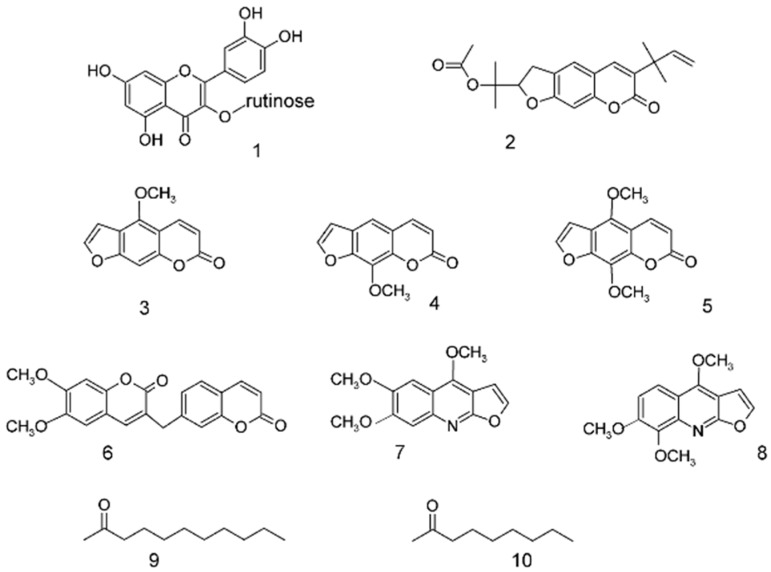
Isolated phytochemicals from rue. **1**: rutin; **2**: rutamarin; **3**: bergapten; **4**: xanthotoxin; **5**: isopimpinellin; **6**: *O*-methyl-daphnoretin; **7**: kokusaginin; **8**: skimmianine; **9**: 2-undecanone; **10**: 2-nonanone.

In this work, we investigated potential taste-active and bioactive compounds from rue grown in the Valcamonica Valley in Northern Italy. We isolated several phytochemicals from aerial parts of the plant (leaves, stems, fruits) by the taste-guided analysis technique. The obtained compounds (Figure 1) belong to different chemical families, including psoralenes, coumarins, alkaloids and volatile ketones from the essential oil.

The compounds were purified and submitted to structural analysis, then tested *in vitro* against a platform of twenty TAS2R bitter taste receptors and four TRP ion channels. The data were compared and are discussed in terms of their relationship to the described taste profile and medicinal properties of this plant.

## 2. Results and Discussion

### 2.1. Isolation of Phytochemicals

*Ruta graveolens* was cultivated at the Università della Montagna (UNIMI) at Edolo (Valcamonica Valley, Lombardy, Italy) in 2012. The aerial parts were harvested in autumn and dried at room temperature. A sample of fresh leaves was frozen and used for essential oil distillation.

Dried leaves, fruits (including pericarps and seeds), stems and seeds were analyzed separately. After extraction with methanol, the extracts were chromatographed over silica gel with different chromatographic techniques; in some cases, purification was obtained using high performance liquid chromatography (HPLC). Compounds **2**–**8** have been found in many parts of the plant in different amounts. Fractions containing complex mixtures of compounds have not been separated. The overall separation scheme to give pure compounds **2**–**8** and the amounts in milligrams of each compound isolated in pure form are described in Supplementary Material (Scheme S1 and Table S1).

Essential oil (EO) was obtained by steam distillation of frozen leaves and was used for *in vitro* assays without further purification. NMR analysis showed the presence of many components, including long chain ketones **9** and **10**, which have been described as constituting more than 90% of EO [12].

The phytochemical analysis of *Ruta graveolens* grown in Valcamonica Valley (Italy) took to identifying several compounds already identified in rue from other regions. A tentative map of the compounds’ distribution in leaves, flowers and fruits was made. During the separation process, we focused our attention on the most important classes of phytochemicals in rue that could be responsible for the bitter taste and/or pharmacological activity related to taste and somatosensory receptors. Rutin **1** was not isolated, since it is commercially available; therefore, a commercial sample was used for *in vitro* assays. Among the furocoumarins, we isolated rutamarin **2** and three psoralenes, **3**, **4** and **5**. Among the alkaloids, we isolated kokusaginin **7** and skimmianine **8**; the resolution of these two compounds was particularly difficult and required HPLC, because they coeluted in every other chromatographic system. 

The total amount of extract was similar for leaves and fruits and slightly lower for stems. The psoralene derivatives (**3**, **4** and **5**) are particularly abundant in fruits, while the quinolinic alkaloids **7** and **8** are almost equally distributed in the aerial parts of the plant.

### 2.2. In Vitro Assays of Rue Phytochemicals on TAS2R and TRP Receptors

As already reported, the sensory profile of rue is characterized by a strong bitter taste of the leaves, sometimes associated with chemesthetic properties, such as pungency. The essential oil, which includes the most volatile compounds, is also bitter and pungent, with a balsamic and sometimes unpleasant odor.

Pure phytochemicals were submitted to *in vitro* assays with a platform of twenty cloned receptors of the bitter taste TAS2R family and four receptors of the TRP family related to the perception of taste and somatosensory sensations. All experiments have been repeated three times, each time with four replicates. For this first screening, compounds have been evaluated at a single dose, where only a qualitative result can be obtained; the agonist or antagonist behavior has been expressed as strong (+++), medium (++), weak (+) or inactive (−). For simplicity, only those bitter taste receptors having reproducible positive assay, namely TASR10, 14 and 49, are reported (Table 1).

**Table 1:** *In vitro* assays of isolated phytochemicals and essential oil from rue.

CPD	TAS2R Assays			TRP Assays			
	TAS2R10	TAS2R14	TAS2R49	TRPA1	TRPM5	TRPM8	TRPV1
**1**	−	−	−	−	−	−	−
**2**	−	−	−	−	+	§	+
**3**	+++	−	−	−	−	−	−
**4**	+	+	++	+	−	−	−
**5**	++	+	−	+	−	−	−
**6**	−	−	−	−	−	−	−
**7**	−	−	−	−	−	−	−
**8**	−	+	−	−	−	−	−
**9**	−	−	−	−	−	−	−
**10**	−	−	−	−	−	−	−
**EO**	−	++	−	−	−	−	−

CPD = compound; numbers are reported as in Figure 1. Screening results have been measured at a single dose and qualitatively reported with the following symbols: agonist activity: +++, strong; ++, medium; +, weak; −, inactive; antagonist activity: §.

For those compounds showing activity on one or more receptor(s), the concentration/response curves were plotted and the EC_50_ calculated. In some cases, the curve did not reach a plateau, and the EC_50_ could only be estimated as a minimal value; these values are indicated with the symbol ≥ (greater than or equal to) in Table 2.

**Table 2:** List of EC_50_ values calculated from the concentration/response analysis performed for all active compounds and the referenced agonist.

TAS2R Referenced Agonists		
Receptor	Compound	EC_50_
TAS2R10	Denatonium Benzoate	8.3 µM
TAS2R10	**3**	2.8 µM
TAS2R10	**4**	≥20.6 µM
TAS2R10	**5**	12 µM
TAS2R14	Aristolochic Acid	2.3 µM
TAS2R14	**4**	≥10.8 µM
TAS2R14	**5**	11.1 µM
TAS2R14	**8**	≥15.8 µM
TAS2R14	**EO**	3.9 mg/L
TAS2R49	Ritanserin	6 µM
TAS2R49	**4**	12 µM
TRPA1	Allyl Isothiocyanate	1.1 µM
TRPA1	**3**	n.d.
TRPA1	**4**	≥19.7 µM
TRPA1	**5**	≥22.8 µM
TRPM5	Carbachol	4.3 µM
TRPM5	**2**	≥26.7 µM
TRPM8	WS3	4.0 µM

Values marked with ≥ are estimated because the dose-response curves did not saturate. n.d. = non detectable.

### 2.3. Rue Phytochemicals and Bitter Taste TAS2 Receptors

**Figure 2:**
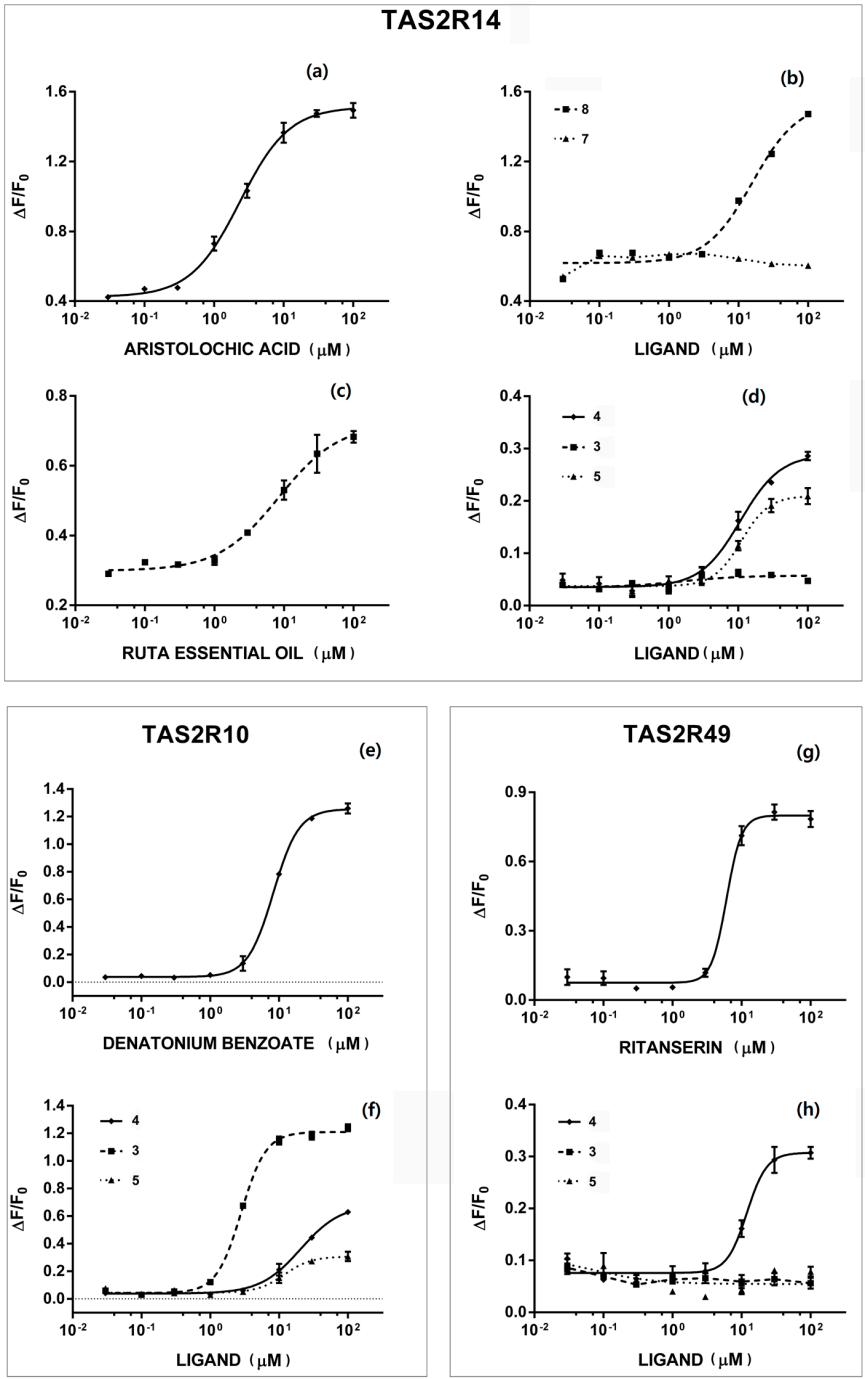
*In vitro* assays on TAS2R receptors of rue phytochemicals and essential oil. Each dose/response curve was performed at least three times using replicates (*n* = 4). TAS2R14: (**a**) aristolochic acid; (**b**) Compounds **7** and **8**; (**c**) ruta essential oil; (**d**) Compounds **3**, **4** and **5**. TAS2R10: (**e**) denatonium benzoate; (**f**) Compounds **3**, **4** and **5**. TAS2R49: (**g**) ritanserin; (**h**) Compounds **3**, **4** and **5**.

The results of *in vitro* assays with TAS2R receptors are shown in Figure 2.

Rutin **1** and the long chain ketones **9** and **10** were inactive in the assays with cloned receptors at the tested concentration. The essential oil of rue is bitter and has a strong disagreeable smell; in *in vitro* assays, it showed a strong and selective response to receptor TAS2R14 which, was not due to Compounds **9** or **10** and, therefore, is likely due to some other minor component still unidentified.

Alkaloids are generally considered to be bitter [27], even if this association is seldom assessed through appropriate sensory analysis or *in vitro* tests. Kokusaginin **7** did not respond *in vitro* to any of the twenty bitter taste receptors, at least at the tested concentration, whereas skimmianine **8** is able to selectively activate receptor TAS2R14.

Compounds **2**–**6** are all structurally related to coumarin, a natural compound responsible for the herbaceous flavor of grass and other vegetables. Coumarin is a known agonist of TAS2R10 and 14 [4]. In our assays, we found that among the coumarin derivatives **2**–**6**, the only inactive compound was the dicoumarin derivative **6**; all of the other compounds responded to some TAS2R receptors, but with very different degrees of selectivity. Bergapten **3** was a selective and strong agonist of TAS2R10, while isopimpinellin **5** also responded to TAS2R14 and xanthotoxin **4** to TAS2R10 and 49. Receptors TAS2R10 and 14 are quite generalist and are broadly tuned by a large number of agonists [2,4]. TAS2R10 in particular is able to accommodate diverse ligands, as demonstrated by a remarkable study using molecular modelling, single point mutagenesis and functional assays [28]. TAS2R10 and 14 usually are activated by the same agonists, while it seems to be uncommon that one of the two and not the other is activated, *i.e.*, it is quite uncommon to find selective agonists for these two receptors; in fact, only cucurbitacin E and erythromycin have been described as selective agonists of TAS2R10, while the only described selective agonist for TAS2R14 is noscapine, an antitussive drug.

The role of TAS2R49 is less described in the literature; to our knowledge, only two synthetic compounds (cromolyn and diphenidol) have been described as non-selective agonists [4]. Therefore, xanthotoxin **4** seems to be the first identified natural compound able to activate this receptor.

### 2.4. Structure-Activity Relationship

A systematic structure-activity relationship requires a large series of derivatives and/or an appropriate model of ligand-receptor interaction, which is not available in the case of rue derivatives. Nevertheless, some qualitative structure-activity relationships can be posited on the basis of existing studies (see below).

Rutin **1** has the structure of quercetin, with a rutinose *O*-glycoside attached in the 3-position. Recently, the structural requirements for bitter taste receptor activation in flavonoids and isoflavonoids have been discussed [2]. Following this paper, it is possible that the presence of hindered substituents, such as the sugar in the 3-position, prevents binding with the receptor; in fact, luteolin (H in 3-position) is active, while quercetin (OH in 3-position) is still active, but with lower efficacy, and rutin **1** (this paper, *O*-rutinose in 3-position) is inactive.

The three furanocoumarins activate TAS2R10 with a decreasing efficacy: **3** > **5** > **4** (Table 2). In the interaction with the TAS2R10 receptor, the position of the methoxyl group seems to be important; in fact, the activity is high when the methoxyl residue is in position 5 (Compound **3**); it decreases when a second methoxyl is added in position 8 (Compound **5**) and decreases further when only this position is occupied (Compound **4**). It would be interesting to compare these results with those obtained by Born and coworkers [28] who identified a putative binding site on TAS2R10 for many different bitter compounds, such as strychnine, parthenolide, denatonium benzoate, cucurbitacin B, santonin, costunolide, papaverine and chloramphenicol; nevertheless, the structures of Compounds **3**, **4** and **5** are quite different, and a comparison is not easy without appropriate molecular modelling tools.

In the case of the TAS2R14 receptor, the role of methoxyl substituents in positions 5 and 8 of the coumarinic aromatic ring seems to be reversed; in fact, the activity follows the order **4** > **5**, while Compound **3** is inactive.

As in many other cases, the presence/absence or the different location of a single substituent can dramatically change the activity: this effect is evident even in the case of isomeric alkaloids **7** and **8**, where the shift of one methoxyl group can totally change the response to TAS2R14.

### 2.5. Rue Phytochemicals and Somatosensory TRP Channels

The presence of certain somatosensory sensations, such as hotness, pungency or coolness, in food plants is often mediated by receptors of the TRP family [1,29]. We tested all isolated compounds with four receptors of the TRP family: TRPA1, an ankyrin receptor associated with the pungency of isothiocyanates; TRPM8, the melastatin receptor responsible for the cooling sensation of menthol and derivatives; the vanilloid receptor TRPV1; and TRPM5, which is involved in the transmission cascade of bitter, sweet and umami perception mediated by GPCR taste receptors.

**Figure 3:**
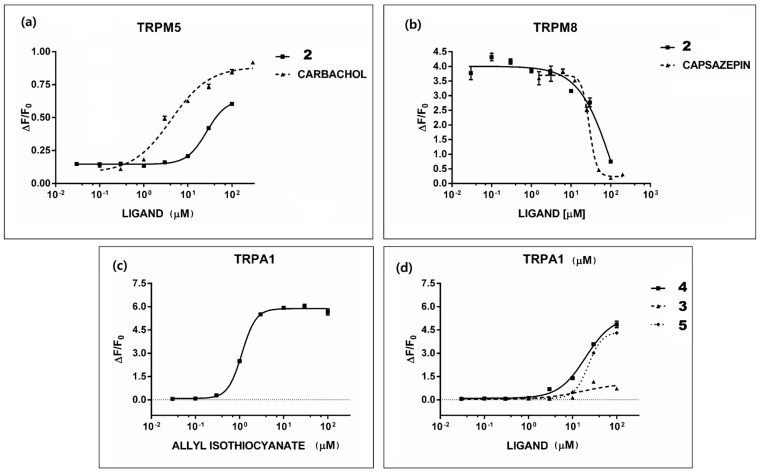
*In vitro* assays with TRP channels. Each dose/response curve was performed at least three times using replicates (*n* = 4). *In vitro* activity of rutamarin **2** and its reference compound capsazepine on (**a**) TRPM5 and (**b**) TRPM8. *In vitro* activity on TRPA1 of (**c**) the reference compound allyl isothiocyanate and (**d**) Compounds **3**, **4** and **5**.

Rutamarin **2** did not activate receptor TRPA1 *in vitro*. On the other hand, Compound **2** activated the TRPM5 ion channel with a curve that is similar to that of the reference agonist carbachol; therefore, a common mechanism of activation could be possible. Rutamarin is also a strong antagonist of TRPM8, as shown in Figure 3.

The IC_50_ value of Compound **2** against TRPM8 calculated from a concentration/response curve was 124 μM, using capsazepine as a reference compound (Figure 3b).

The TRPM8 receptor is overexpressed in some organs, such as liver and prostate, and is highly upregulated in prostate cancer cells, where it may be involved in tumor cell proliferation [30,31]. TRPM8 inhibitors therefore represent a potential new approach to the treatment of prostate cancer, and the search for such compounds, especially of natural origin, is therefore very active. Recently, the sphingoid leucettamols have been identified as potent TRPM8 inhibitors of marine origin [32]. While we were preparing this manuscript, a paper was also published reporting that the alkaloid voacangine, extracted from the roots of an African plant, is a potent TRPM8 inhibitor [33].

Our report that rutamarin **2** has this kind of activity is then a further example of a natural TRPM8 inhibitor from the plant kingdom. In this case, the plant is quite accessible, and the structure of Compound **2** is much simpler than that of voacangine; therefore, this kind of skeleton seems attractive as a potential lead for pharmacological studies.

The fact that Compound **2** is also an agonist of TRPV1 and Compounds **4** and **5** are agonists of TRPA1 is remarkable. In fact, both of these channels and TRPM8 are not only acting as “gustative ” receptors, but also play an important role in nociception and are implicated in neuropathic and inflammatory pain [34,35,36]. The obtained results suggest that some of the isolated compounds can also exert some antinociceptive activity *in vivo*, a hypothesis that would be in agreement with the curative properties described for rue in traditional and folk medicine and also supported by some recent studies [21].

Finally, compounds able to activate bitter taste receptors and TRPs are very often associated with irritation and repulsion effects and/or some toxicity in insects, invertebrates and other animals, and they are traditionally used as repellents and pesticides. Therefore, our findings about the activity of rue derivatives could also be related to the very well-known use of rue extracts as insect and animal repellents. Rue, together with many other bitter food plants, is confirmed to be a very good reservoir of bioactive compounds with specific biological targets that can be usefully applied for pharmaceutical, agri-food and other applications.

## 3. Experimental Section

Reagents were of commercial grade purity, and solvents were dried by a standard procedure. Column chromatography was carried out on 220–240 mesh silica gel using the flash methodology or alumina; thin-layer chromatography was obtained on Merck precoated silica gel 60 F254 plates (Merck, Kenilworth, NJ, USA), and the spots were visualized by UV at 254 and 365 nm or stained with suitable reagent development. All compounds were checked for purity by HPLC using a Varian SD200 liquid chromatograph system with an Altima RP18 column (250 mm length, 4.6 mm ID, 5 μ, Alltech, (Alltech-Grace, Columbia, MD, USA) NMR spectra were recorded on Bruker AMX-300 and Bruker Avance-600 instruments (Bruker Biospin GmbH, Rheinstetten, Germany), using TMS as an internal standard; *J* values are given in Hertz.

LC-MS analyses were recorded with a Bruker Daltonics APEX II ICR-FTMS instrument (Bruker) using the ESI ionization mode. Optical rotation was recorded at 549 nm (sodium D line) in cuvettes with a 10-cm cell length and a 1-mL capacity on a Perkin-Elmer 141 polarimeter (Perkin-Elmer, Waltham, MA, USA). UV spectra were registered on a Perkin-Elmer Lamba 2 UV/VIS spectrophotometer (Perkin-Elmer).

### 3.1. Plant Material

*Ruta graveolens* was cultivated at the Università della Montagna (UNIMI) at Edolo (Valcamonica Valley, Italy) in 2012. The aerial parts were harvested in autumn and dried at room temperature. A sample of fresh leaves was frozen and used for essential oil distillation.

### 3.2. Extraction and Isolation

Dried leaves; fruits (including pericarps and seeds); stems and seeds were analyzed separately. Portions of 100 g of each were ground and extracted with methanol (leaves: 500 mL; fruits: 350 mL; stems: 500 mL) at room temperature for 48 h. The extracts were filtered and concentrated *in vacuo*. We obtained extracts E1 (leaves; 12.63 g; 126 mg/g dry weight), E2 (fruits; 13.61 g; 136 mg/g dry weight) and E3 (stems; 0.09 g; 94 mg/g dry weight). They were chromatographed over silica gel under gradient conditions with a mixture of hexane and ethyl acetate as the eluent; starting with a ratio of 7:3 (*v*/*v*); then changing to ethyl acetate 100%; and then to pure methanol.

Three different chromatographic techniques were used: flash chromatography on silica gel (FC), column chromatography on alumina (CCA) or preparative thin layer chromatography (PTLC); in some cases, purification was obtained using high performance liquid chromatography (HPLC). Compounds **2**–**8** have been found in many parts of the plant in different amounts. Fractions containing complex mixtures of compounds have not been separated. The overall separation process to give pure Compounds **2**–**8** and the amounts in milligrams of each compound isolated in pure form are described in Supplementary Material, Scheme S1 and Table S1.

Essential oil was obtained by standard steam distillation of frozen leaves (40 g of leaves in 1 L of water for a total time of 1 h) and was used for *in vitro* assays without further purification. NMR analysis showed the presence of many components, including long chain ketones **9** and **10**, which have been described as constituting more than 90% of the oil [12]. For further analysis, commercial samples of these two compounds (Sigma-Aldrich, Saint Louis, MO, USA) were used. Rutin **1** is also a commercial product (Sigma-Aldrich); therefore, we did not isolate this compound from rue, and we used a commercial sample for *in vitro* assays.

### 3.3. Spectroscopic Analysis of Compounds

Compounds **2**–**5**, **7** and **8** had spectroscopic data (NMR, MS) consistent with what has been reported in the literature: (+)-**2**, rutamarin (14882-94-1), [37]; **3**, bergapten (484-20-8); **4**, xanthotoxin (298-81-7) [38]; **5**, isopimpinellin (482-27-9) [39]; **7**, kokusaginin (4842-08-2) [40]; **8**, skimmianine (83-95-4) [20].

Compound **6** has already been described in the literature [41]; however, the reported NMR spectrum was recorded using chloroform and trifluoroacetic acid as solvents, and the signals we obtained in pure chloroform were slightly different; therefore, the structure was confirmed using ^1^H- and ^13^C-NMR spectra and heteronuclear multiple bond correlation (HMBC) experiments. The assignment of carbon was made on the basis of heteronuclear single quantum coherence spectroscopy (HSQC) (Supplementary Material, Figure S2).

The structure of Compound **6** was also confirmed by mass spectrometry: LC-MS (ESI) *m*/*z* = 389.06339 calculated for [M + Na]^+^ (C_20_H_14_O_7_Na^+^).

### 3.4. In Vitro Assays with TAS2R and TRP Receptors

#### 3.4.1. Compounds and References

For the biological tests, several compounds were purchased as references from different suppliers (Sigma Aldrich, ChromaDex, Irvine, CA, USA, and Tocris Bioscience, Bristol, UK) and dissolved in assay buffer (130 mM NaCl, 5 mM KCl, 1 mM MgCl_2_, 2 mM CaCl_2_, 5 mM NaHCO_3_ and 20 mM HEPES, pH 7.4) or in a mixture of assay buffer and DMSO, never exceeding a final DMSO concentration of 0.5% (v/v) to avoid toxic effects and possible artefacts on transfected cells. The complete list of compounds used as references is reported in Table 3.

#### 3.4.2. Vectors and cDNAs

All human TAS2R and G15Gi1 cDNAs were produced as reported in Bassoli *et al.* [42] cDNAs for human TRPA1-M5-M8-V1-V4 were cloned into a pcDNA6 expression vector (Invitrogen-Thermo Fisher, Waltham, MA, USA).

**Table 3:** List of TAS2Rs receptors and reference compounds used as agonists.

TAS2R Referenced Agonists		
Receptor	Compound	Concentration
TAS2R1	Menthol	300 µM
TAS2R3	Chloroquine	10 mM
TAS2R4	Colchicine	3 mM
TAS2R5	Phenanthroline	300 µM
TAS2R7	Chloroquine	10 mM
TAS2R8	Chloramphenicol	300 µM
TAS2R9	Ofloxacin	3 mM
TAS2R10	Denatonium Benzoate	300 µM
TAS2R13	Denatonium Benzoate	3 mM
TAS2R14	Aristolochic Acid	10 µM
TAS2R16	Salicin	3 mM
TAS2R38	Phenylethyl Isothiocyanate	300 µM
TAS2R39	Denatonium Benzoate	3 mM
TAS2R40	Cinchinone	100 µM
TAS2R43	Aristolochic Acid	1 µM
TAS2R44	Aristolochic Acid	10 µM
TAS2R46	Strychnine	10 µM
TAS2R47	Denatonium Benzoate	30 µM
TAS2R49	Ritanserin	100 µM
TAS2R50	Andrographolide	30 µM

The reported concentrations are the highest used for each receptor.

#### 3.4.3. Cell Culture and Transfection

All experiments were performed using HEK293T cells (ATCC).

For TRP platform experiments, cells were cultured in MEM/EBSS medium (ECB2071L EuroClone, Milan, Italy) plus 10% (*v*/*v*) fetal bovine serum (FBS) (26400-044 Gibco, Karlsruhe, Germany) and 1% (*v*/*v*) Pen/Strep 10K/10K stock (17-602E Lonza, Basel, Switzerland). Stable cell lines were generated overexpressing each TRP channel, and clones were selected with 500 µg/mL of G418 (ant-gn-5 Invivogen, Karlsruhe, Germany). To analyze TRP receptor responses, cells were plated at a concentration of 20,000 cells/well in 384-well plates (GR-4330 Twin Helix srl, Milan, Italy). Cellular responses were measured 24 h after seeding.

For the bitter platform experiments, cells were cultured, transfected and analyzed as previously described [42].

#### 3.4.4. Calcium Imaging Analysis and Quantification

For the bitter platform experiments, data analyses were performed as previously described [42].

For TRPA1-M8-V1-V4 experiments, a solution of Fluo8-NW calcium-sensitive dye (36316 AAT BioQuest, Sunnyvale, CA, USA) was prepared using the assaybuffer and diluting the kit components 1:2 according to the manufacturer’s instructions. Twenty-four hours after seeding, cells were incubated one hour at RT, loaded with 40 µL of Fluo8-NW solution for each well and incubated again for one hour at RT before data acquisition.

For TRPM5 experiments, cells were loaded 24 h after seeding with a Membrane Potential Dye (R8034 MDC) solution in assay buffer according to the manufacturer’s instructions and incubated at 37 °C for 1 h before acquisition. All experiments were performed using a fluorometric imaging plate reader (FLIPR^TETRA^, Molecular Devices equipped with ICCD Camera from Stanford Photonics, INC, (Palo Alto, CA, USA) and kinetics were visualized with Molecular Devices’ Screenworks 3.0 (Sunnyvale, CA, USA).

For the calculation of concentration-response curves, responses were calculated as the difference between maximal and minimal relative fluorescence unit (RFU) values in a selected time window (11–60 s) and were normalized to the basal well fluorescence (Time Point 1, before compound injection) in order to compensate for differences in cell density (∆F/F0). All of the experiments have been repeated three times, each time with four replicates. All of the results are averages of at least four replicates. Raw data are available in Supplementary Material, Figures S3–S6.

All calculations and plots were made using Microsoft Excel 2010 (Redmond, WA, USA) and GraphPad Prism 6.0 (La Jolla, CA, USA).

## Supplementary Materials

Supplementary materials can be accessed at: http://www.mdpi.com/1420-3049/20/10/18907/s1.
