# Supplementary material for: Phytochemicals from Ruta graveolens Activate TAS2R Bitter Taste Receptors and TRP Channels Involved in Gustation and Nociception
Source: Molecules. 2015 Oct 16;20(10):18907–22. doi: 10.3390/molecules201018907 (PMC6331789; doi:10.3390/molecules201018907)
Supplement: Supplementary file 1 [file molecules-20-18907-s001.pdf]

Supplementary Material

1. Chromatographic Separation of Phytochemicals

Dried leaves, fruits (including pericarps and seeds), stems and seeds were analyzed separately. Portions of 100 g each were ground and extracted with methanol (leaves: 500 mL, fruits: 350 mL; stems: 500 mL) at room temperature for 48 h. The extracts were filtered and concentrated *in vacuo*. We obtained extracts E1 (leaves, 12.63 g, 126 mg/g dry weight), E2 (fruits, 13.61 g, 136 mg/g dry weight) and E3 (stems, 0.09 g, 94 mg/g dry weight). They have been chromatographed over silica gel in gradient conditions using as the eluent a mixture of hexane and ethyl acetate starting with a ratio of 7:3 (*v*/*v*), to ethyl acetate 100% and then pure methanol.

Three different chromatographic techniques were used: flash chromatography on silica gel (FC), column chromatography on alumina (CCA) or preparative thin layer chromatography (PTLC); in some cases, the purification was obtained using high performance liquid chromatography (HPLC). The overall separation process to give pure Compounds **2**–**8** is described in Scheme S1.

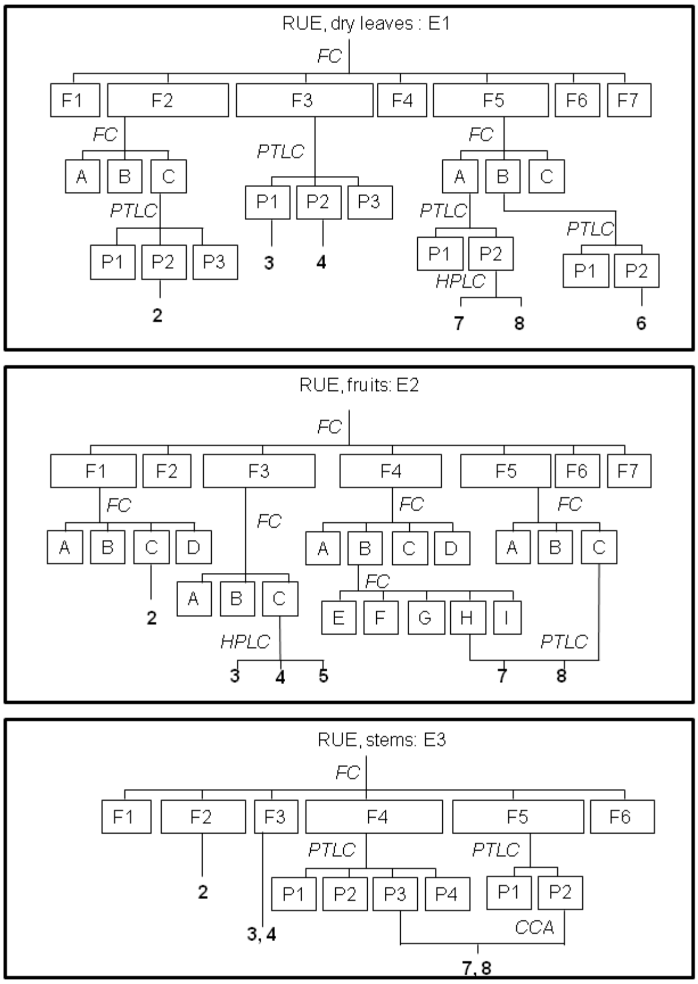

Scheme S1. Chromatographic separation of Compounds **2**–**8**. FC, flash chromatography, silica gel; PTLC, preparative thin layer chromatography; HPLC, high performance liquid chromatography, RP 18; CCA, column chromatography, alumina.

Table S1. Weight of pure Compounds **2**–**8** (mg) isolated from rue.

CPD	E1 (Leaves)	E2 (Fruits)	E3 (Stems)
**2**	37	44	21
**3**	25	67	14
**4**	57	52	
**5**	-	20	-
**6**	5	-	-
**7**	12	6	7
**8**	7		

2. NMR Data for Compound 6

Table S2. ^{1}H and ^{13}C chemical shifts (ppm), long-range H-C correlations (HMBC) and coupling constants *J* in Hz for compound **6** in $CDCl_3$.

Number	δ ^{1}H (Moltelicity, *J* in Hz)	δ ^{13}C	HMBC
2		157.3	
3		137.6	
4	7.42 s	128.7	107.6 (C8)<br>137.6 (C3)<br>147.7 (C7)<br>157.3 (C2)
4a		96.2	
5	6.91 s	99.8	147.7 (C7)<br>147.2 (C8a)
6		152.3	
7		147.7	
8	6.83 s	107.6	128.7 (C4)<br>147.7 (C7)<br>147.2 (C8a)<br>152.3 (C6)
8a		147.2	
2′		160.3	
3′	6.33 d (*J* = 9.56)	114.7	104.8 (C8′)<br>113.5 (C6′)
4′	7.67 d (*J* = 9.56)	142.9	155.3 (C8a′)<br>160.3 (C2′)
4a′		113.9	
5′	7.46 d (*J* = 8.59)	129.2	159.3 (C7′)<br>155.3 (C8a′)<br>142.9 (C4′)
6′	7.00 dd (*J* = 2.43 and 8.59)	113.5	
7′		159.4	
8′	6.96 d (*J* = 2.43)	104.8	113.9 (C4a′)<br>155.3 (C8a′)<br>160.3 (C2′)
8a′		155.3	
6-OCH_3 *	3.95 s	56.41	152.3 (C6)
7-OCH_3 *	3.91 s	56.41	147.7 (C7)

*: might be interchangeable.

1D and 2D NMR spectra suggest the presence of two coumarin moieties. The ^{1}H-NMR and HMBC spectra shown two doublets at 6.33 and 7.67 ppm (*J* 9.56 Hz) characteristic of the H-3′ and H-4′ of the coumarin ring and three aromatic protons as a doublet at 7.46 ppm (*J* 8.59 Hz), a doublet of doublet at 7.00 ppm (*J* 2.43 and 8.59 Hz) and doublet at 6.96 ppm (*J* 2.43 Hz) assigned to H-5′, H-6′ and H-8′. All of these protons belong to the same coumarin moiety. Based on HMBC correlations, three singlets at 6.91, 6.83 and 7.42 were assigned to H-5, H-8 and H-4, respectively, of the second coumarin ring. This substitution pattern suggests a C3, C-7′ linkage between the two moieties.

3. Calcium Traces for MOCK and TAS2Rs Transfected Cells

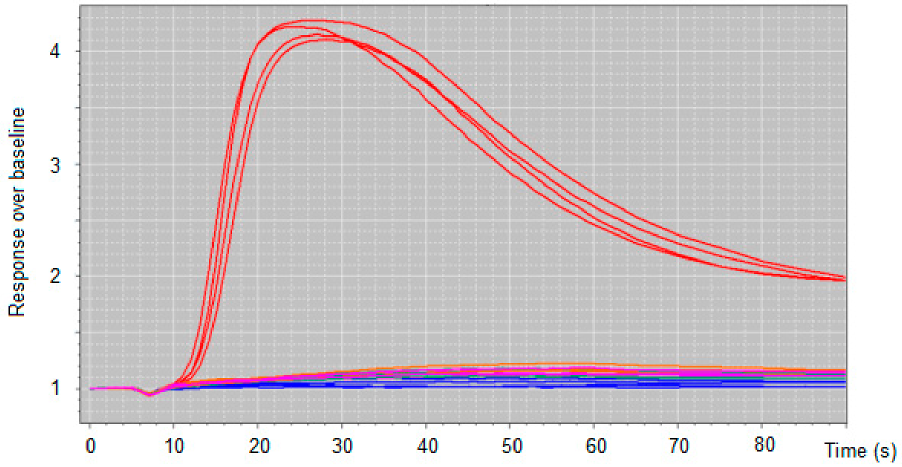

Figure S3. Raw traces for MOCK transfected cells treated with endogenous beta adrenergic receptor agonist (isoproterenol, 10 µM), denatonium benzoate (TAS2R10 agonist, 300 µM), aristolochic acid (TAS2R14 Agonist, 10 mM), ritanserin (TAS2R49 agonist, 100 µM) and Tyrode's buffer (negative control).Isoproterenol = red; denatonium benzoate = green; aristolochic acid = yellow; ritanserin = pink; Tyrode's buffer = blue.

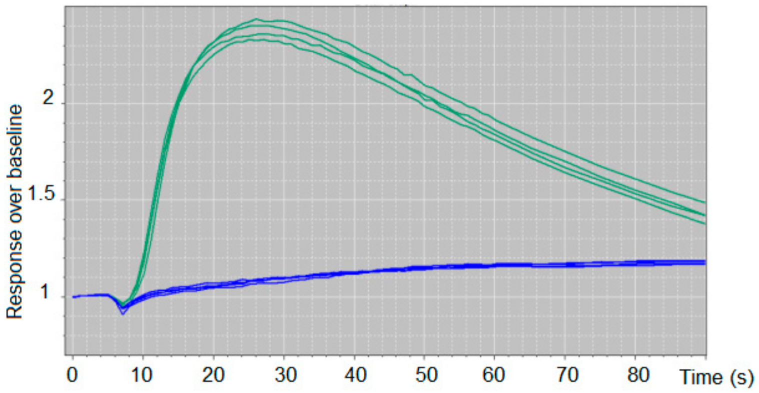

Figure S4. Raw traces for TAS2R10 transfected cells treated with denatonium benzoate (TAS2R10 agonist, 300 µM) and Tyrode's buffer (negative control). Denatonium benzoate = green; Tyrodes's buffer = blue.

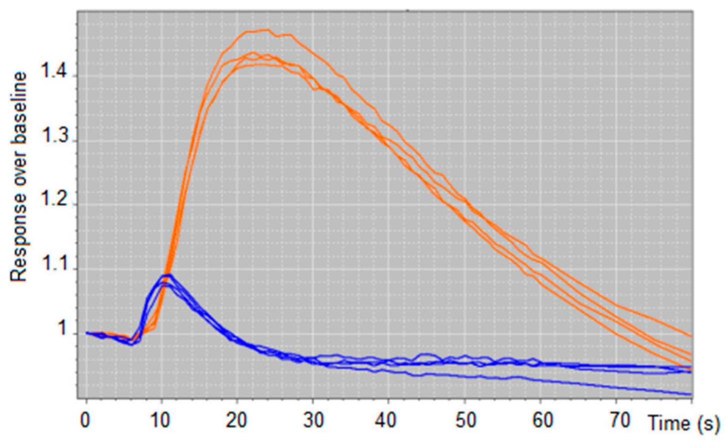

Figure S5. Raw traces for TAS2R14 transfected cells treated with aristolochic acid (TAS2R14 agonist, 10 mM) and Tyrode's buffer (negative control). Aristolochic acid = yellow; Tyrode's buffer = blue.

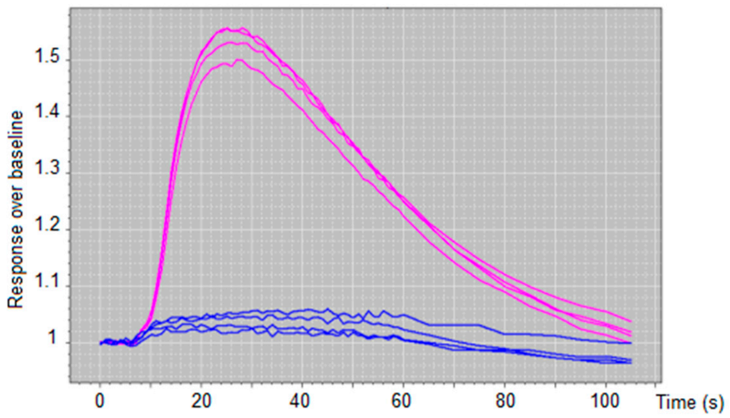

Figure S6. Raw traces for TAS2R49 transfected cells treated with ritanserin (TAS2R49 agonist,100 μM) and Tyrode's buffer (negative control). Ritanserin = pink; Tyrode's buffer = blue.
